# Ultra-Sensitive Simultaneous Detection of Dopamine and Acetaminophen over Hollow Porous AuAg Alloy Nanospheres

**DOI:** 10.3390/nano14131131

**Published:** 2024-06-30

**Authors:** Menghua Li, Xinzheng Liu, Changhui Sun, Xiaorong Cao, Yuanyuan Zhang, Linrui Hou, Hongxiao Yang, Caixia Xu

**Affiliations:** 1 Department of Chemistry, Qilu Normal University, Jinan 250011, China; liuxz@163.com (X.L.); sunch@qlnu.com (C.S.); xrcao@qlnu.com (X.C.); 2 School of Chemistry and Chemical Engineering, School of Materials Science and Engineering, University of Jinan, Jinan 250022, China; 17862903610@163.com (Y.Z.); chm_xucx@ujn.edu.cn (C.X.)

**Keywords:** AuAg HPNSs, electrochemical sensor, dopamine, acetaminophen, simultaneous detection

## Abstract

Hollow porous AuAg nanospheres (AuAg HPNSs) were obtained through a simple solvothermal synthesis, complemented by a dealloying strategy. The hollow interior, open pore voids, and integral interconnected skeleton shell in AuAg HPNSs are beneficial for providing sufficient electrolyte diffusion and contacts, abundant active sites, and efficient electron transport. This specific structure and the favorable alloy synergism contribute to the superior electrocatalytic activity toward dopamine (DA) and acetaminophen (AC). AuAg HPNSs show high sensitivity, good selectivity, excellent sensing durability, and outstanding repeatability for amperometric assays of AC and DA. In particular, the AuAg-based sensors achieve effective ultrasensitive simultaneous analyses of AC and DA, exhibiting the characteristics of the wide linear range and low detection limit. With their prominent electrocatalytic activity and simple preparation methods, AuAg HPNSs present broad application prospects for constructing a highly responsive electrochemical sensing system.

## 1. Introduction

The precise and fast monitoring of dopamine (DA) and acetaminophen (AC) in human metabolic fluid has always fascinated countless researchers. DA is extensively distributed in the central nervous system, hormones, and cardiovascular system of mammals, exerting a crucial function in information transmission and learning processes [1]. A multitude of health disorders, including Alzheimer’s disease, Parkinson’s disease, and Schizophrenia, have been found to be highly associated with the abnormal level of DA [2]. As an analgesic and antipyretic, AC is a broadly used non-prescription medication to treat cold and fever symptoms, relieve toothache, headache, muscle pain, and postoperative pain. Meanwhile, it serves as a potential prodrug for the treatment of drug-resistant cancers [3,4]. Nevertheless, an overdose of AC can cause adverse effects, including minor metabolic organ damage, pancreatic disease, or even fatal hepatotoxicity and nephrotoxicity [4]. Moreover, DA and AC are both nitrogenous aromatic compounds with analogous structural and chemical properties and are concurrently present in the actual biological systems [5]. Both in vivo and in vitro studies have indicated that the dosage of AC affects the concentration levels of DA [6]. The high dosage intake of AC could affect the DA levels and thus interfere with the DA analysis [7]. According to the above, the simultaneous quantification of DA and AC in the actual samples holds significant importance.

At present, a variety of innovative, highly sensitive, and selective methods for the precise measurement of DA and AC are constantly being reported, such as spectrophotometric for AC [8], mass spectrometry for DA [9], thermometric titrimetry for AC [10], surface plasmon resonance for DA [11], and high-performance liquid chromatography for AC [12]. However, these assays are usually time-consuming and require strict experimental environment with complicated sample pretreatments. Compared with the above methods, the electrochemical analysis of AC and DA has become an excellent method characterized by its rapid response, easy construction, high reproducibility, high sensitivity, and miniaturization potential [13,14]. Moreover, both DA and AC are aromatic compounds, possessing similar chemical structures and properties. And the oxidation peak potentials of DA and AC commonly overlap with each other on some traditional electrode surfaces [15]. By virtue of their large active surface area as well as excellent catalytic activities, nanomaterials can dramatically improve electron transfer efficiency to achieve much more sensitive responses and realize the simultaneous detection of AC and DA [16,17,18,19,20]. Therefore, developing highly efficient nanostructured materials is highly desirable for separating the electrochemical signals of AC and DA. Until now, copper porphyrin-exfoliated graphene [6], ferrocene thiolate Fe_3_O_4_@Au nanoparticle-modified copper nanowire nanocomposites [21], Al_2_O_3_-supported palladium nanoparticle [22], NiO_x_- and CuO_x_-decorated graphene materials [23], and so on have been reported to construct electrochemical sensors for achieving the simultaneous detection of DA and AC efficiently. Although the successful separation of the electrochemical signals of the DA and AC has been accomplished, the relative complex preparation method and complicated construction limit their extensive use for the concurrent sensing of DA and AC. Continuous endeavors are still needed to exploit highly active electrode materials with simple and controllable preparation in order to further promote practical application.

Engineering the microstructure and components of the nanocomposites represents the valuable protocol for the improvements of sensing performances. It is well reported that hollow porous nanomaterials with hollow interiors and porous shells could facilitate the mass transport, promote the electrolyte flowing, and provide more active sites [24,25]. Among the various nanomaterials, Au-based structures have been proven to possess unique electrocatalytic activity and high sensitivity for many active species [26]. First, the Au-based materials have superior biocompatibility and can be used for the specific detection of multiple target substances [27]. Furthermore, the formation of Au-based alloys by introducing other transition metals usually generates a preferable synergism compared with their single counterparts [19]. As a sensitive catalyst, pure Ag is rarely used owing to its relatively poor stability. However, the AuAg alloy can integrate the advantages of Au and Ag as well as deliver new merits in terms of excellent conductivity, superior sensitivity, and high stability [26]. Consequently, the regulation of AuAg alloy nanomaterials with superior sensing performances by convenient and scalable methods is remarkable [28].

In the current work, we aimed to fabricate hollow porous AuAg nanospheres (AuAg HPNSs) using one simple solvothermal method followed by a dealloying strategy. Uniform hollow AuAg alloy nanospheres were first fabricated by one solvothermal process with high Ag content. The partial dissolution of Ag in an HNO_3_ solution for hollow AuAg alloy nanospheres further generates rich pore voids on the hollow shells as well as enlarges the hollow interiors. By virtue of the specific hollow nanoporous microstructure and the favorable alloy synergism, AuAg HPNSs respond quickly with sensitive simultaneous detection toward DA and AC. On account of the exceptional catalytic capabilities of AuAg HPNSs and the specialized nature of the preparation method, AuAg HPNSs hold promising application prospects in electrochemical detection.

## 2. Materials and Methods

### 2.1. Materials and Characterization

The reactants dopamine (DA) and acetaminophen (AC) were acquired from Sigma-Aldrich (Shanghai, China). Hydrogen tetrachloroaurate trihydrate (HAuCl_4_·3H_2_O) was obtained commercially from Aladdin Industrial Inc (Shanghai, China). Ethylene glycol (C_2_H_6_O_2_), silver nitrate (AgNO_3_), nitric acid, polyvinylpyrrolidone (PVP), ammonia solution, nafion (5 wt.%), and ethanol were sourced from Sinopharm Chemical Reagent Co., Ltd. (Shanghai, China). The phosphate-buffered saline (PBS, pH 7.4) solution was prepared using KH_2_PO_4_ and Na_2_HPO_4_. Ultra-pure water with a resistivity of 18.2 MΩ cm was employed throughout all experiments. 

### 2.2. Synthesis of AuAg HPNSs

The preparation AuAg HPNSs was conducted utilizing a refined solvothermal method [29]. In a typical procedure, 0.4 g PVP was dissolved in 10 mL ethylene glycol under sonication to form a homogeneous solution. Then, 0.1 g AgNO_3_, 1.5 mL HAuCl_4_·3H_2_O (0.1 g mL^−1^ in (CH_2_OH)_2_), and 240 μL ammonia solution were added sequentially under continuous stirring. The uniform solution was then introduced into the 20 mL Teflon-lined autoclave and maintained at 200 °C for 4 h. After the autoclave was naturally cooled to the room temperature, the resulting precipitate was retrieved by centrifugation, and washed 3 times with water and ethanol, respectively. The resultant AuAg HPNSs were acquired through etching the precursor in 14 M HNO_3_ solution for a period of 15 min at ambient temperature, followed by extensive rinsing with ultra-pure water.

### 2.3. Characterization Tests

The morphological and microstructural characteristics of the samples were obtained using high-resolution transmission electron microscope (HRTEM, JEOL JEM-2100F, JEOL Ltd., Japan) and field-emission scanning electron microscope (SEM, HITACHIS-4800, HITACHI Ltd., Japan). The crystal phase of the AuAg alloy was acquired on X-ray diffraction (XRD, Bruker D8 advanced, Bruker Group, Germany) with Cu Kα radiation. The chemical states of AuAg HPNSs were analyzed via X-ray photoelectron spectroscopy (XPS) through a Thermo Scientific ESCALAB 250 X-ray photoelectronic spectrometer, employing a monochromatized Mg Kα X-ray as the excitation source (Thermo Scientific, MA, USA). The BET method was conducted on the Micromeritics Tristar II 3020 (Micromeritics Corporate, GA, USA) instrument to obtain the specific surface area and the pore size distribution. CHI 760E electrochemical workstation from Shanghai CH Instruments Co., China was utilized for all electrochemical measurements. A conventional three-electrode system was applied with the glassy carbon electrode (GCE) as the working electrode, accompanied by a mercurous sulfate electrode serving as the reference electrode, and a platinum plate as the counter electrode. All potentials noted in this work were normalized to the reversible hydrogen electrode (RHE).

### 2.4. Fabrication of the AuAg HPNS-Modified GCE 

The preparation of the catalyst ink involved mixing 1.0 mg AuAg HPNSs, 1.0 mg carbon powder, 200 μL ethanol, and 200 μL nafion solution (0.5 wt.%) with the aid of sonication. Subsequently, 4 μL of electrocatalyst ink was deposited on GCE as a working electrode.

### 2.5. Simultaneous Detection of DA and AC in Human Serum

To validate the utility of the detection method, the standard addition technique was employed for the simultaneous determination of DA and AC in human serum. The serum samples were sourced from Shandong Tumor Hospital. Prior to analysis, the serum samples were diluted 100 times with 0.1 M PBS solution, followed by the addition of specific concentrations of DA and AC. The concentrations of DA and AC were determined using the differential pulse voltammetry (DPV) on a AuAg HPNS-based sensor. The assay method was evaluated by determining the recovery and relative standard deviation (RSD).

## 3. Results and Discussion

### 3.1. Structure and Morphology of AuAg HPNSs

Zero-dimensional nanoparticles, characterized by high specific surface area, can be fixed on the GCE more robustly by a nafion binder. The solvothermal method represents one of the highly efficient techniques to fabricate alloy nanoparticles with controllable morphology and components. We adopted the solvothermal method in ethylene glycol solvent with mild reaction conditions to first fabricate hollow AgAg nanoparticles, where a part of Ag atoms was then selectively removed in order to construct open pore voids on the shell of hollow particles.

**Figure 1:**
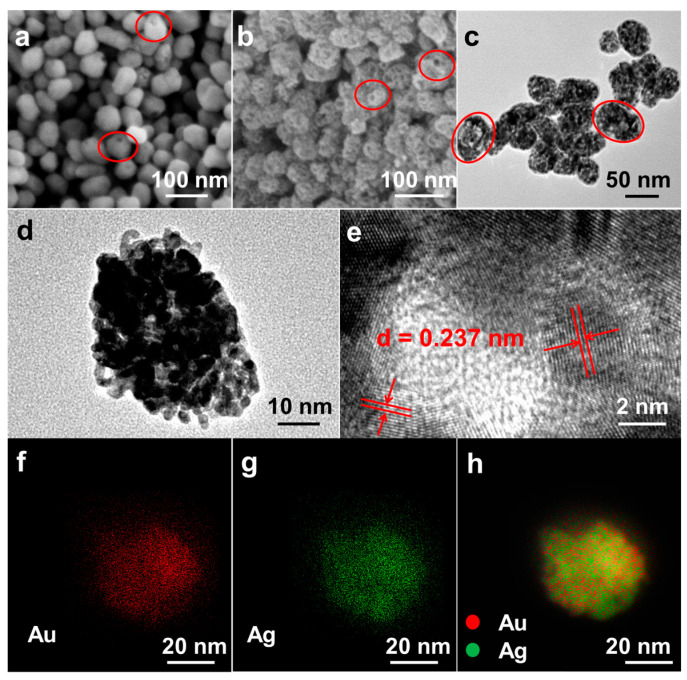
(**a**) SEM image of the initial AuAg nanoparticles after the solvothermal treatment, (**b**) SEM, (**c**,**d**) TEM, (**e**) HRTEM, and (**f**–**h**) elemental mapping images of the AuAg HPNSs.

**Figure 2:**
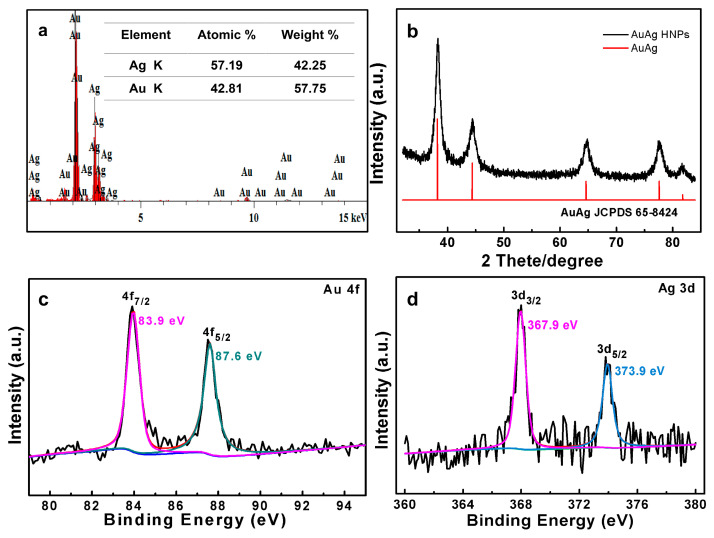
(**a**) EDS data of the AuAg HPNSs, (**b**) the XRD pattern of the AuAg HPNSs. XPS data of Au 4f (**c**) and Ag 3d (**d**) of the AuAg HPNSs.

The microstructures of hollow AuAg alloy particles and the de-alloyed AuAg sample were revealed by electron microscopy. Uniform AuAg nanospheres with a diameter of about 50 nm can be distinguished after the solvothermal operation (Figure 1a). In addition, the apparent open channels running through the entire particle can be observed as denoted by the red circles, which demonstrated the formation of hollow nanospheres with the continuous shells and interior voids. The EDS analysis in Figure S1 indicates that the AuAg precursor alloy has the composition of Au_12.26_Ag_87.74_. Its high Ag content provides substantial basis to create high porosity through a de-alloying process. After being etched in nitric acid solution (~14 M) for 15 min, tiny pores penetrating the shell of the hollow nanospheres were clearly found, which resulted from the selective dissolution of Ag atoms (Figure 1b). Interestingly, the hollow nature and original nanospherical shape of the de-alloyed sample remained almost unchanged, while the interior voids enlarged owing to the removal of part of Ag atoms. The detailed structure information of the AuAg alloy was further revealed by TEM image. As indicated in Figure 1c, the distinct color contrast between the black interconnected skeleton and internal bright areas further demonstrated the formation of hollow AuAg alloy nanospheres with tiny pores uniformly distributed in the nanoshells. The typical thickness of a porous nanoshell is around 8 nm. Notably, due to the suitable projection angle of the TEM view, the open channels of some nanospheres can also be found, as denoted by the red circles. The EDS results in Figure 2a indicate that the composition of the final AuAg HPNSs is Au_43_Ag_57_, testifying that the selective dissolution of Ag produces rich open pores on the hollow shell. The high-magnification TEM image depicted in Figure 1d offers further evidence that the AuAg alloy nanospheres possessed the continuous nanoscaled framework with hollow interior and abundant pores with a narrow ligament of ~3 nm. The hollow porous nature and robust interconnected nanoporous skeleton structure endow AuAg HPNSs with the greatest advantages, such as rich reactive sites and smooth pathway for mass and electron transport during the operation of electrochemical sensors. The pore size distribution of AuAg HPNSs is demonstrated in Figure S2. The BJH desorption test indicates the pore diameter of AuAg HPNSs mainly located in the range of 2.5 to 15 nm.

The corresponding HRTEM image in Figure 1e shows a highly ordered lattice stripe of ~0.237 nm, which is just between the (111) interplanar spacing of Ag (0.238 nm) and Au (0.235 nm). Au and Ag are in miscible phase because of the same fcc crystal structures and the comparable lattice stripe [30]. Therefore, the lattice fringes can be ascribed to the (111) plane structure of the AuAg alloy [30]. As shown in Figure 1f–h, the elemental mappings reveal a homogeneous distribution of Au and Ag elements across the nanospheres, illustrating the successful synthesis of AuAg alloy. The experimental observations above expressly testify that the scalable fabrication of uniform hollow porous AuAg nanospheres can be achieved through the solvothermal method and de-alloyed strategy. It is remarkable that the selective corrosion of a portion of Ag atoms from AuAg hollow nanospheres can generate rich open pores penetrated through the entire shell.

The crystal structure of the obtained sample was identified utilizing XRD with reference to standard diffraction patterns for comparative analysis (Figure 2b). The diffraction pattern of AuAg HPNSs exhibits one set of five diffraction peaks positioned at approximately 38.3°, 44.4°, 64.6°, 77.6°, and 81.8°, which is suggestive of the fcc AuAg alloy (JCPDS No. 65-8424). XPS was also carried out to reveal the chemical valence states in AuAg HPNSs with the core level spectra of Au 4f and Ag 3d shown in Figure 2c,d. The binding energy around 83.9 and 87.6 eV in Figure 2c is indicative of Au 4f_7/2_ and Au 4f_5/2_ [31]. The two peaks at 367.9 and 373.9 eV in the Ag 3d spectra (Figure 2d) correspond to the spin orbit of Ag 3d_5/2_ and Ag 3d_3/2_, respectively. The energy discrepancy between the two peaks is ~6.0 eV, illustrating the metallic state of the Ag element in AuAg alloy [32]. The XRD patterns and XPS data further manifest the successful formation of AuAg alloy. 

### 3.2. Electrochemical Analysis of DA and AC on the AuAg HPNSs

**Figure 3:**
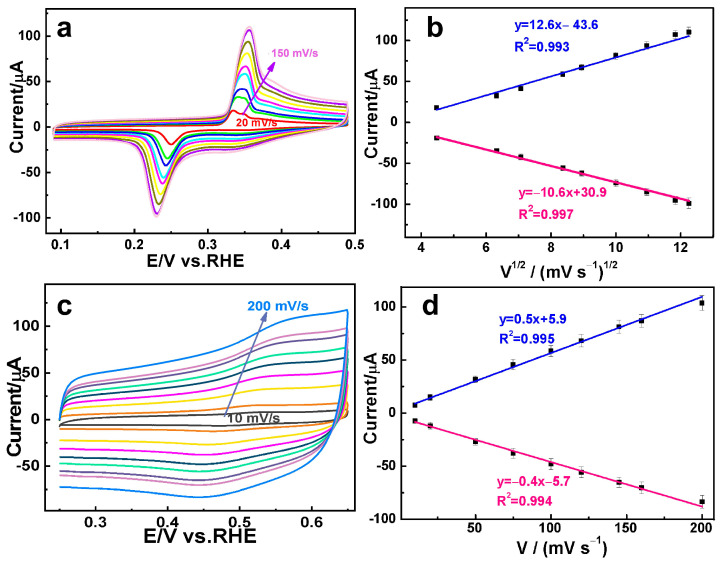
CV curves for (**a**) DA and (**c**) AC oxidation over AuAg HPNSs in PBS with different scan rates. (**b**) Plots of anodic peak currents vs. the square root of the scan rate of DA, and (**d**) the peak currents vs. scan rates of AC.

AuAg HPNSs possess a hollow porous structure composed of nanoscaled skeleton, hollow interior, and rich pore channels. The unique structure facilitates the unlimited mass transport, abundant active sites, and unblocked electrolyte diffusion in the electrocatalytic process. Such ideal hollow porous architecture is conducive to construct a robust sensing platform to differentiate multiple compounds. Consequently, it is intriguing to assess the electrocatalytic capability of AuAg HPNSs for DA and AC. The cyclic voltammetric (CV) curves with 0.01 mM DA in 0.1 M PBS solution (pH 7.4) at different scan rates are shown in Figure 3a. A pair of well-defined redox peaks at 0.35 and 0.23 V was clearly observed. It is well reported that DA is firstly converted to dopaminequinone (DAQ) through an initial oxidation during the cyclicvoltammetric scan process. DAQ is inclined to generate intracellular cyclization through 1, 4-Michael reaction, which is further oxidized to Leucodopaminechrome (LDAC) and then to dopaminechrome (DAC) [33]. The electrooxidation mechanism of DA on the modified electrode are provided in Figure S3 [34]. The low DA concentration, the thermodynamics and kinetics changes during anodic scanning may result in a relatively rapid and large reduction in DAQ, which additionally affect the depletion of DAQ to produce redox peaks [35]. The anodic current associated with DA oxidation increases in proportion to the scan rate while the peak potential undergoes a slightly positive shift accompanied by the similar onset potential. A direct proportionality between the peak current and the square root of the scan rate is presented in Figure 3b (linear equation: *y* = 12.6 *x* − 43.6, *R*^2^ = 0.993, and *y* = −10.6 *x* + 30.9, *R*^2^ = 0.997), suggesting that the electrochemical oxidation of DA on AuAg HPNSs is a diffusion-controlled process [36].

The electrocatalytic response of AuAg HPNSs to the oxidation of AC was also investigated by varying the scan rate. As shown in Figure 3c, the anodic oxidation peak appeared at 0.55 V, associated with a corresponding cathodic counterpart peak at 0.45 V during the sweep process. The voltammetric reaction of AC over the AuAg HPNSs correlates with the transformation of AC to N-acetyl-p-benzoquinone-imine, followed by the subsequent reduction of N-acetyl-p-benzoquinone-imine to AC [37]. The chemical equations for the oxidation mechanism of AC can be observed in Figure S4 [5]. As depicted in Figure 3d, the peak currents of AC over AuAg HPNSs exhibit a linear dependence on the scan rate (linear equation: *y* = 0.5 *x* + 5.9, *R*^2^ = 0.995; *y* = −0.4 *x* − 5.7, *R*^2^ = 0.994), indicative of an adsorption-controlled process in the electrochemical reaction [3]. Both DA and AC oxidation over AuAg HPNSs exhibit an excellent linear dependence of voltammetric peak currents on the scan rate. The excellent linear relationship and the relatively wide interval between the oxidation peak potentials of DA and AC endow AuAg HPNSs with the capability of simultaneous detection of DA and AC.

**Figure 4:**
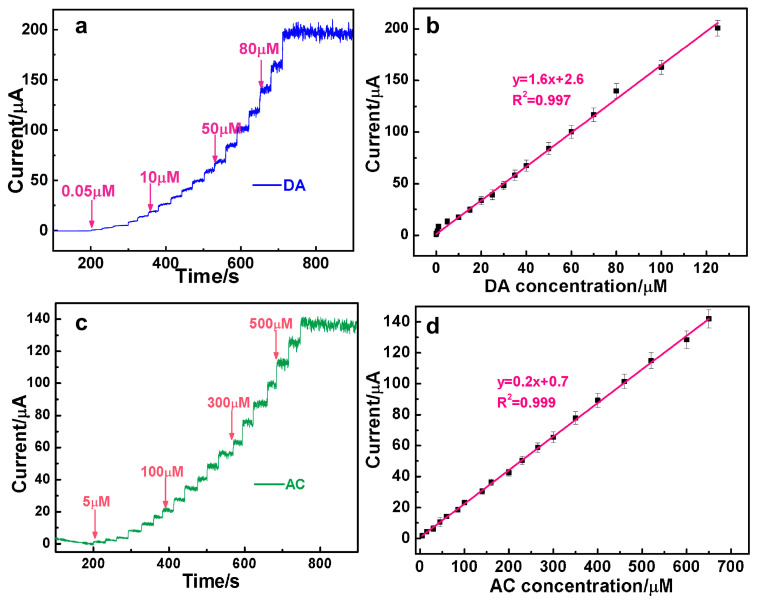
Sequential amperometric responses of AuAg HPNSs to incremental addition of DA at 0.35 V (**a**) and AC at 0.55 V (**c**) into PBS solution. The linear calibration plots of current against the concentrations of DA (**b**) and AC (**d**).

The typical amperometric responses of the AuAg-HPNS-based sensor to the continuous addition of DA and AC were recorded, which could reflect the sensitivity and detection range of the as-constructed sensor. As illustrated in Figure 4a, upon the addition of DA at the specified potential of 0.35 V, an obvious current response was observed and approached the maximum stable current within 0.5 s. The peak current increases linearly with DA concentration within the range of 0.05–130 μM with the limit of detection as low as (S/N = 3). The linear equation (*y* = 1.6 *x* + 2.6, *R*^2^ = 0.997) shows the direct linear correlation between the oxidation peak currents and the variation in concentrations of DA (Figure 4b). Similarly, the current response generated on the AuAg HPNSs with each introduction of AC at 0.55 V achieves the maximum steady-state current within 1.5 s (Figure 4c). Figure 4d presents the linear responses of AuAg HPNSs to AC within 5–650 μM (linear equation: *y* = 0.2 *x* + 0.7, *R*^2^ = 0.999) with a detection limit of 0.42 μM (S/N = 3). 

AuAg HPNSs offer high-sensitivity fast response, a large linear concentration range, and a reduced detection limit in detecting DA and AC. The sensing capabilities of the developed sensor were evaluated against the previous reports presented in Table S1 [21,38,39,40,41,42,43,44,45]. AuAg HPNSs present much better or comparable catalytic performances with those of reported sensors, demonstrating that AuAg HPNSs have great application potential to construct sensors. 

### 3.3. The Durability and Reproducibility for DA and AC Determination over AuAg HPNSs

**Figure 5:**
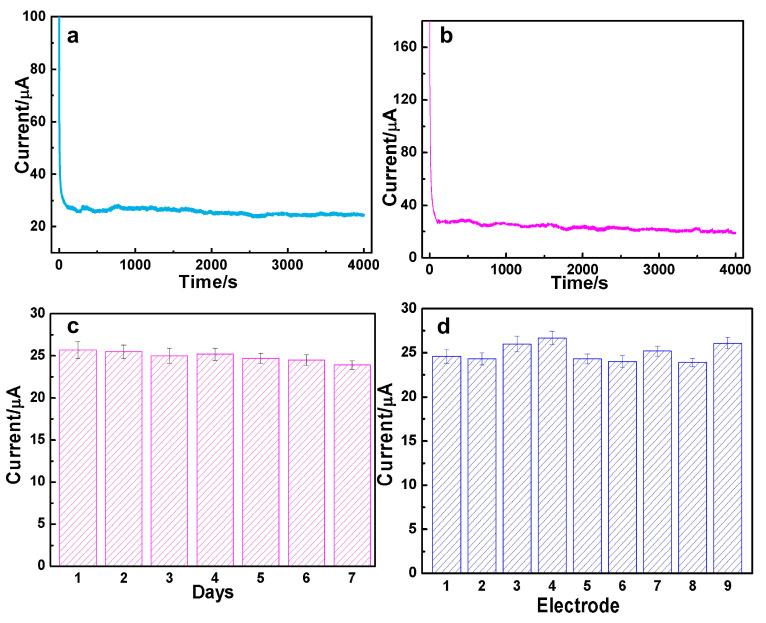
Sensing stability of AuAg-HPNS-based sensors in the PBS solution with (**a**) 0.01 mM DA at 0.35 V and (**b**) 0.1 mM AC at 0.55 V for successive 4000 s. (**c**) Long-term durability of AuAg-HPNS-based sensors for DA measurement for 7 days. (**d**) Reproducibility of the AuAg-based sensors with 9 different electrodes towards AC detection.

In order to assess the enduring electrocatalytic capabilities of AuAg HPNSs, a potentiostatic method was carried out to test its steady-state behavior. It can be seen that 98.2% of the initial current responding to 0.01 mM DA was remained after continuously running for 4000 s at 0.35 V (Figure 5a). Meanwhile, only 7.3% activity decay in response to 0.1 mM AC was observed after 4000 s at 0.55 V (Figure 5b). The chronoamperometry data declare that AuAg HPNSs have outstanding sensing stability towards DA and AC.

The long-term stability of AuAg-HPNS-based sensors for DA detection was also evaluated once a day for 7 consecutive days (Figure 5c). The low relative standard deviation, 2.49%, proves its highly long-term catalytic durability. Additionally, the reliability of the sensors was measured by examining the current responses of nine individual electrodes towards 0.1 mM AC at 0.55 V under the same operating condition. Nine electrodes exhibit similar response currents, with an acceptable relative standard deviation of 8.79% (Figure 5d). The data above suggest that AuAg-HPNS-based sensors have superior durability and good reliability for AC and DA determination.

### 3.4. The Simultaneous Quantification of DA and AC over AuAg HPNSs

**Figure 6:**
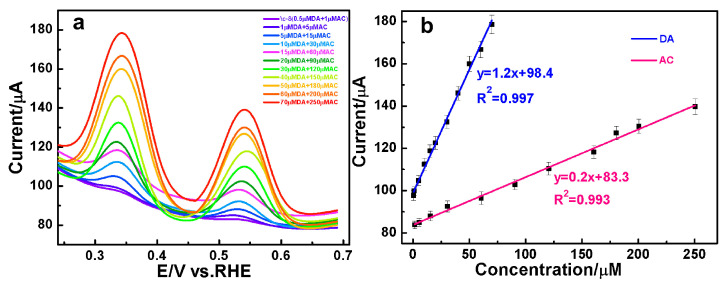
(**a**) DPV responses of AuAg HPNS-modified GCE toward varying concentrations of DA and AC in 0.1 M PBS. (**b**) Calibration plots of peak currents vs. analyte concentrations.

Encouraged by the outstanding amperometric responses of the constructed sensors, the DPV was performed to test the concurrent measurement of DA and AC over AuAg HPNSs. As presented in Figure 6a, two distinct volt-ampere peaks, ascribed to the oxidation of DA (0.35 V) and AC (0.55 V), can be resolved clearly. Most importantly, the current in response of DA and AC oxidation is linearly dependent on the concentration. The direct proportional relation of anodic peak currents versus DA concentrations were observed with the linear expression of *y* = 1.2 *x* + 98.4, *R*^2^ = 0.997 at concentrations between 0.5 and 70 μM. Meanwhile, the proportionality observed at concentrations between 1 and 250 μM for AC adheres to the linear equation *y* = 0.2 *x* + 83.3, *R*^2^ = 0.993. The DPV files prove that the AuAg-HPNS-based sensor is highly competent in concurrent analysis of DA and AC. To facilitate a clearer understanding of the detection capabilities of the AuAg-HPNS-based sensor, the linear equations, the domain of linear concentration ranges, and the associated data for the simultaneous detection of DA and AC via DPV, as well as their individual amperometric detection are presented in Table S2.

### 3.5. The Simultaneous Analysis of DA and AC in Human Serum

The feasibility of the AuAg-HPNS-based sensor for the simultaneous determination of DA and AC in the real sample was assessed in human serum using the standard addition method. Prior to detection, the human serum sample was treated by a 100-fold dilution with 0.1 M PBS. The results manifest that the AuAg-HPNS-modified electrode could detect DA and AC with acceptable recovery and RSD (Table 1), stating the reliable and utilizable feature of the sensor for determining DA and AC in actual samples.

### 3.6. The Selectivity for DA and AC Detection over AuAg HPNSs

**Figure 7:**
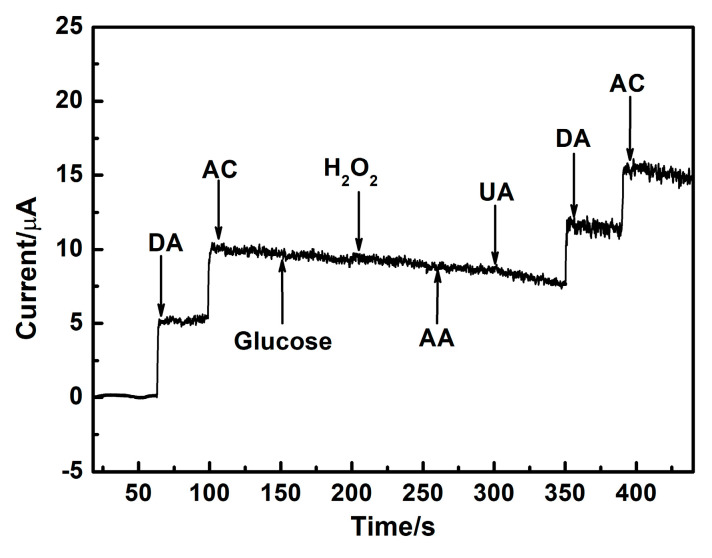
Amperometric signals upon the continuous introduction of 5 μM DA, 50 μM AC, 0.5 mM glucose, 0.2 mM H_2_O_2_, 0.5 mM AA, 0.5 mM UA, 5 μM DA, and 50 μM AC on AuAg-HPNS-based sensors in a stirring PBS solution at 0.45 V.

The anti-interference of the AuAg-HPNS-based electrochemical sensor also serves as a crucial parameter for assessing its application prospect. The compounds that may coexist with DA and AC, such as glucose, H_2_O_2_, AA, and UA, were added successively to investigate the anti-interference of the AuAg-HPNSs. The amperometric responses of the sensor based on the AuAg HPNSs exhibit evident signals towards the DA and AC (Figure 7), while the successive injection of 0.5 mM glucose, 0.2 mM H_2_O_2_, 0.5 mM AA, and 0.5 mM UA produced tiny signals at 0.45 V. The electrochemical signal of interfering substances generated on AuAg HPNSs can be ignored compared with those caused by DA and AC at 0.45 V. According to the results above, it is conclusive that the AuAg-HPNS-based sensors exhibit outstanding anti-interference towards coexisting electroactive species and possess the potential foreground of practical application in DA and AC detection.

In summary, AuAg HPNSs show the superior sensing performances toward DA and AC with features including rapid response, a broad linear range, minimal detection limit, strong anti-interference, reliable reproducibility, and enduring stability, which may originate from their specific microstructure as well as the preferable alloy synergism. First, the abundant active sites from the internal and outside surfaces of hollow porous shells can furnish massive active sites to significantly boost the electrocatalytic activity towards DA and AC. Second, the rich smooth channels widely distributed throughout the AuAg HPNSs facilitate the fluent mass diffusion and sufficient contact of active sites with reactants. Third, the robust integral interconnected network shell can offer a highly conductive skeleton to promote a rapid electron transfer in an electrocatalytic process. Finally, the synergistic effect from a AuAg alloy can generate the excellent electrocatalytic activity and thus help to further achieve highly sensitive and selective sensing. 

## 4. Conclusions

In the current work, hollow porous AuAg alloy nanospheres with integrated nanoporous skeleton structures were successfully fabricated through a simple solvothermal method and dealloying procedure. Characterized by the hollow interior, high porosity, and alloy synergism, the AuAg HPNSs show excellent eletrocatalytic activity for DA and AC oxidation. The AuAg-HPNS-based analysis platform exhibits high sensitivity, broad linear range, dependable selectivity, and durable stability towards DA and AC detection. The outstanding sensing performance mainly derives from the abundant reactivity sites from the hollow porous structure, fluent diffusion, and sufficient contact of the electrolyte from the rich pores, high electron conductivity from the interconnected ligament, and favorable synergistic alloying effect of the AuAg alloy. More importantly, AuAg-HPNS-based sensors present outstanding capability for an efficient concurrent measurement of DA and AC in biological samples. AuAg HPNSs hold promising prospects in excellent electrochemical sensor construction and their practical application for the simultaneous quantification of DA and AC. 

## Figures and Tables

**Table 1:** Simultaneous recovery of DA and AC in human serum samples.

No. of Serum Sample	Added (μM)		Found (μM)		RSD (%) (n = 3)		Recovery (%)	
	DA	AC	DA	AC	DA	AC	DA	AC
1	15	60	15.6	59.6	3.14	3.06	104.0	99.3
2	40	150	40.9	148.6	3.08	3.17	102.2	99.1
3	60	200	62.2	203.5	2.53	2.95	103.7	101.8

## Data Availability

Data will be made available on request from the authors.

## Supplementary Materials

The following supporting information can be downloaded at: https://www.mdpi.com/article/10.3390/nano14131131/s1. Figure S1. EDS data of the nano AuAg precursor alloy; Figure S2. (a) The N_2_ adsorption and desorption isotherm and (b) the pore size distribution of AuAg HPNSs; Figure S3. The possible oxidation mechanism of DA on the AuAg HPNSs-modified electrode; Figure S4. The proposed mechanism for the oxidation of AC on the AuAg HPNSs-modified electrode. Table S1. Comparison of the analytical parameters for the determination of DA and AC over AuAg HPNSs-based sensors by electrochemical method with some other sensors reported in literatures; Table S2. The detection capabilities of the AuAg-HPNSs-based sensor for DA and AC.
