# Supplementary material for: Ultra-Sensitive Simultaneous Detection of Dopamine and Acetaminophen over Hollow Porous AuAg Alloy Nanospheres
Source: Nanomaterials (Basel). 2024 Jun 30;14(13):1131. doi: 10.3390/nano14131131 (PMC11243617; doi:10.3390/nano14131131)
Supplement: Supplementary file 1 [file nanomaterials-14-01131-s001.zip › nanomaterials-3057116-supplementary.pdf]

Supporting information

Ultra-Sensitive Simultaneous Detection of Dopamine and Acetaminophen over Hollow Porous AuAg Alloy Nanospheres

Menghua Li [1,*], Xinzheng Liu [1], Changhui Sun [1], Xiaorong Cao [1], Yuanyuan Zhang [2], Linrui Hou [2,*], Hongxiao Yang [2,*] and Caixia Xu [2]

1 Department of Chemistry, Qilu Normal University, Jinan 250011, China; liuxz@163.com (X.L.); sunch@qlnu.com (C.S.); xrcao@qlnu.com (X.C.)

2 School of Chemistry and Chemical Engineering, School of Materials Science and Engineering, University of Jinan, Jinan 250022, China; 17862903610@163.com (Y.Z.); chm_xucx@ujn.edu.cn (C.X.);

*Correspondence: limenghua668@126.com (M.L.); mse_houlr@ujn.edu.cn (L.H.); chm_yanghx@ujn.edu.cn (H.Y.)

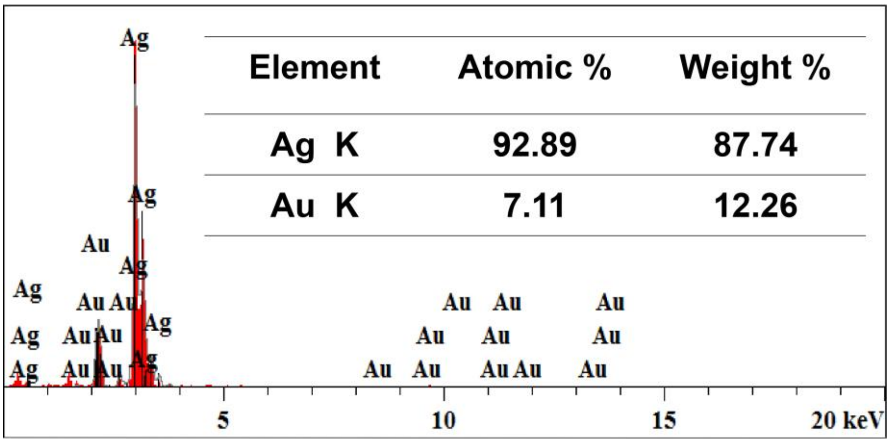

Figure S1. EDS data of the nano AuAg precursor alloy.

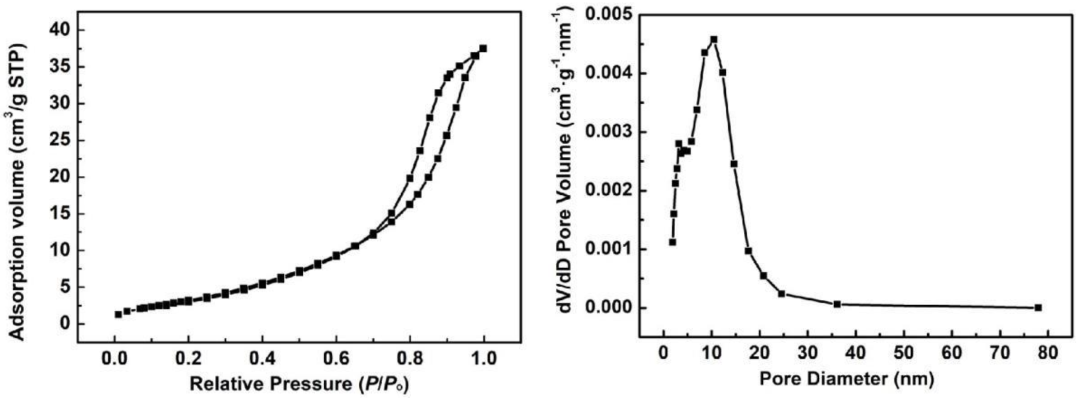

Figure S2. (a) The N_2 adsorption and desorption isotherm and (b) the pore size distribution of AuAg HPNSs.

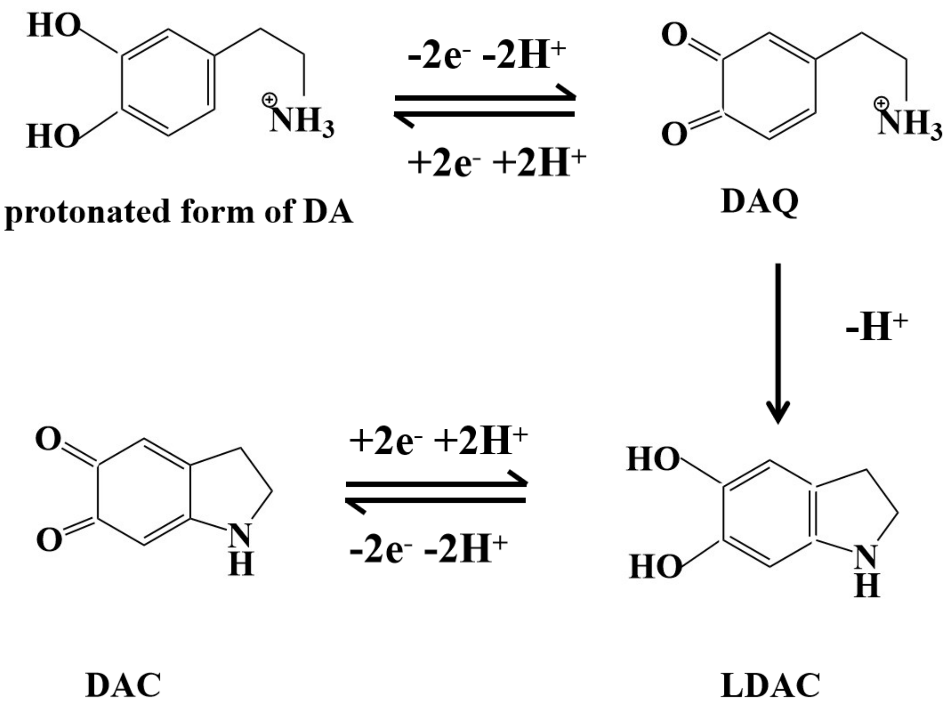

Figure S3. The possible oxidation mechanism of DA on the AuAg HPNSs-modified electrode (dopamine is present in its protonated form in neutral solution[34])

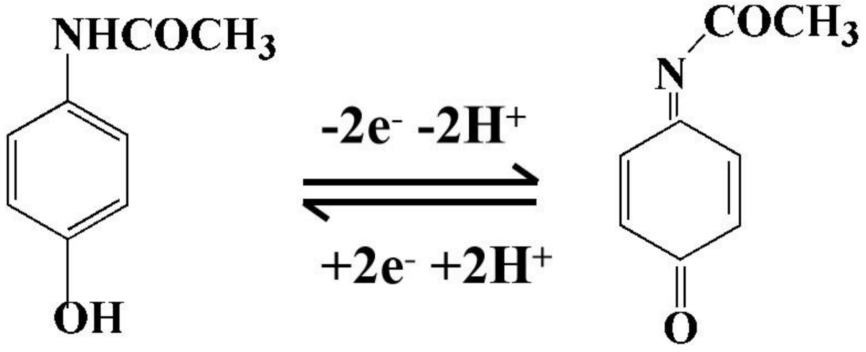

Figure S4. The proposed mechanism for the oxidation of AC on the AuAg HPNSs-modified electrode

Table S1. Comparison of the analytical parameters for the determination of DA and AC over AuAg HPNSs-based sensors by electrochemical method with some other sensors reported in literatures.

Materials	Liner range (μM)			Detection limit (μM)			Ref.
	DA	AC	Others	DA	AC	Others	
Fe_3O_4@Au-S-Fc/GS -chitosan [a]	0.5~50	0.3~ 250	Uric acid: 1~300	0.1	0.05	0.2	[21]
Fc-S-Au/CNC/ Graphene [b]	0.4~ 645	0.6~ 482	Uric acid: 0.9~1440	0.05	0.1	0.12	[38]
Cu-MOFs/MWCN T-Au@Ag [c]	0.6~ 300	1~500	—	0.23	0.082	—	[39]
TCPP-Sa/CPE [d]	0.4~ 10.3	1~90	Tyrosine: 3~51	0.1	0.2	0.7	[40]
f-MWCNTs [e]	3~200	3~300	—	0.8	0.6	—	[41]
PSNSB/CPE [f]	0.05~ 120	0.033~ 158	Ascorbic acid: 2.5~1050	0.00245	0.00536	0.0186	[42]
MWCNTs-NHNPs - MCM-41/GCE [g]	1.5~45 and 70~350	0.2~20 and 20~220	Indomethaci n: 0.8~40 and 60~160	0.15	0.11	0.31	[43]
OMWCNT-GCE [h]	83.3~ 312.5	116.7~ 437.5	Tryptophan: 166.7~625.0	—	—	—	[44]
AuNPs@TCnA/ GN [i]	0.5~150 and 0.3~1000	0.5 ~ 120	—	0.1	0.1	—	[45]
AuAg HPNSs	0.05~130	5~650	—	0.03	0.42	—	This work

a: Fe_3O_4@Au-S-Fc/GS-chitosan: phenylethynyl ferrocene thiolate (Fc-SAc) modified Fe_3O_4@Au NPs coupling with graphene sheet/chitosan (GS-chitosan);

b: Fc-S-Au/CNC/graphene/GCE: thiol functional ferrocene derivative (Fc-SH) stabilized Au NPs/carbon dots nanocomposite (Au/CNC) coupling with graphene modified glassy carbon electrode;

c: Cu-MOFs/MWCNT-Au@Ag: copper porphyrin metal organic frameworks (Cu-MOFs) and Au@Ag core-shell nanoparticle decorated multiwalled carbon nanotubes;

d: TCPP–Sa/CPE: carbon paste electrode modified with clay and porphyrin;

e: f-MWCNTs: acid functionalized multi-wall carbon nanotubes;

f: PSNSB/CPE: PbS nanoparticle Schiff base-modified carbon paste electrod;

g: MWCNTs-NHNPs-MCM-41/GCE: a multiwalled carbon nanotubes, nickel(II) hydroxide nano-particle and MCM-41 molecular sieve modified glassy carbon electrode;

h: OMWCNT-GCE: an oxadiazole derivative MWCNT (multi-walled carbon nanotubes) modified glassy carbon electrode;

i: AuNPs@TCnA/GN: gold nanoparticle (AuNPs) modified by thiolated calix[n]arene (TCnA, n = 4, 6, 8) were anchored on graphene nanosheets (GN) by p-p stacking interaction between TCnA molecules and GN.

Table S2. The detection capabilities of the AuAg-HPNSs-based sensor for DA and AC.

Test condition	The linear equation	R^2	The concentration range	The limit of detection
Individual amperometric detection of DA	$y = 1.6\,x + 2.6$	0.997	0.05-130 μM	0.03 μM
Individual amperometric detection of AC	$y = 0.2\,x + 0.7$	0.999	5-650 μM	0.42 μM
The simultaneous quantification of DA	$y = 1.2\,x + 98.4$	0.997	0.5-70 μM	0.28 μM
The simultaneous quantification of AC	$y = 0.2\,x + 83.3$	0.993	1-250 μM	0.51 μM
